# Mapping historical diaries into knowledge graphs: a multi-granular approach

**DOI:** 10.1007/s42803-026-00119-x

**Published:** 2026-03-09

**Authors:** Sepideh Alassi

**Affiliations:** Digital Humanities Lab (DHLab), University of Basel, Basel, Switzerland

**Keywords:** Digital scholarly edition, Annotation, RDF, Knowledge graph, Ontologies, Historical diaries, Citation

## Abstract

This article presents a methodological framework for representing historical diaries as multi-layered, interactive, region-based RDF knowledge graphs, enabling precise and granular modeling of manuscripts, pages, textual regions, and annotations. It identifies five significant challenges in scholarly digital editions and demonstrates how these can be addressed through a framework that supports segment-level text representation, targeted annotation, and interoperability across editions, languages, and manuscript witnesses. This framework incorporates a segment-level citation system with resolvable, timestamped identifiers, ensuring reproducibility, stable referencing, and reliable scholarly citation over time. To describe the suggested methodologies and functionalities, this paper provides examples from digital editions of Jacob Bernoulli’s *Meditationes* and *Reisbüchlein*, highlighting the potential of RDF ontologies to capture complex editorial layers, including transcriptions, translations, and scholarly commentary.

## Introduction

Scholarly Digital Editions (SDEs) are dynamic, interactive environments that allow users to explore textual variants, annotations, and hyperlinks, thereby enabling new interpretive approaches beyond linear reading (O’Sullivan et al., 2025; Driscoll & Pierazzo, 2016; Gabler, 2010). For decades, many SDEs have relied on TEI/XML,^1^ the standard markup language for structuring textual data in the digital humanities. TEI/XML underpins projects such as *The Newton Project*^2^ and *HallerNet*,^3^ but it faces persistent challenges, notably overlapping markup (Stäcker, 2020). While useful, XML- and HTML-based editions also struggle to capture the systematic, semantic links between textual components. Recent work on so-called “next-generation markup languages” has proposed more expressive schema- and grammar-based solutions to make such complex textual structures computationally tractable (Haentjens-Dekker et al., 2025).

Knowledge graphs (KGs) and *Resource Description Framework* (RDF)^4^ offer a stronger framework for representing SDEs. RDF represents entities as resources with unique *Internationalized Resource Identifier*s (IRIs), relates them through properties, and formalizes meaning via ontologies (Allemang & Hendler, 2012, 6–8). This allows interoperability, reasoning, and integration across repositories. RDF-based SDEs, such as *LetterSampo*,^5^ the *Anton Weber*^6^ and *Carl Maria von Weber*^7^ editions, the *Bernoulli–Euler Online* (BEOL)^8^ correspondence, and *Tolstoy Semantisized* (Bonch-Osmolovskaya et al., 2019), enable semantic search and user-friendly exploration without requiring SPARQL knowledge (Alassi & Rosenthaler, 2024). Despite these advantages, challenges remain: metadata representation, handling transcription layers, managing overlapping markups, designing interfaces that balance usability and depth, and ensuring persistent citation. Recent work on graph-based text modeling, such as the Applied Text as Graph (ATAG) approach (Kuczera, 2025), has further demonstrated how representing text as an addressable network of nodes can enhance transparency, annotation depth, and citation in digital scholarly editions, underscoring a broader shift toward process-oriented and computationally explicit editorial models.

In this paper, I will outline an RDF-based framework to address five main challenges of SDEs.

### **First**,** metadata representation**

TEI typically embeds metadata in transcription headers, which can reduce interoperability (Schmidt, 2014). RDF allows metadata to be modeled as linked resources, enhancing flexibility. For instance, in BEOL and LetterSampo, transcriptions are RDF resources linked to metadata, such as the editor’s identity and transcription date. More granular modeling, down to paragraph or marginal note level, can ensure accurate attribution and metadata allocation.

### **Second**,** transcription layers**

Digital editions typically present one or a few variations of a specific document, such as diplomatic, critical, or normalized editions, and may also include different translations. Printed editions typically offer a transcription version, whereas TEI/XML often embeds multiple renditions with inline tags, such as < del> and < add>, within a transcription file. Inline renditions cause parsing difficulties and complicate the editing process. These kinds of SDEs would require complex XSLT rules to convert renditions into an HTML presentation for the web, a process that can be slow. Storing transcription layers separately would eliminate these issues, simplifying editing, since users and programmers could work with one layer at a time (Schmidt, 2014). In the model proposed in this paper, segments (e.g., paragraphs) can contain multiple transcription layers, each of which can be independently studied, presented, and cited. This approach allows flexible and multi-layered representation.

### **Third**,** markup complexity**

Overlapping or nested markups, such as italicized phrases partly struck through, are challenging to represent in TEI/XML. Modifying them often requires rewriting entire files. Moreover, heavily marked-up texts are poorly suited for machine processing. Schmidt (2014) argues for separating markup from transcription to improve compatibility with natural language processing (NLP). RDF editions can implement standoff markup, representing markup as independent nodes linked to text ranges. This enables easy markup updates, markup-based queries (e.g., retrieving all occurrences of a specific phrase in bold characters), and interoperability with machine learning algorithms for named entity recognition (NER), part-of-speech (POS) tagging, or topic modeling.

### **Fourth**,** web presentation**

SDEs often risk overwhelming readers with hyperlinks and tools. While printed editions emphasize readability and orientation through pagination (Sondheim et al., 2016), digital editions prioritize data richness. Yet, as Mangen (2008, 410) warns, excessive hyperlinks fragment attention. Balancing readability with data exploration is crucial. This paper proposes dynamic, multi-level data modeling and a web infrastructure for interactive data retrieval, allowing users to view facsimiles, transcriptions, and annotations at chosen levels (document, page, segment). This design simulates print reading while preserving access to enriched data.

### **Fifth**,** persistent citation**

The fluidity of SDEs complicates the reliability of referencing (Bleier, 2021). Permalinks and PIDs exist but are inconsistently applied in SDEs. RDF enables fine-grained, persistent identifiers for all components, including documents, pages, paragraphs, and marginal notes, supporting precise and verifiable citations. This granularity allows scholars to reference the exact segment analyzed, ensuring scholarly rigor.

To explain the framework proposed in this paper, I present examples from two multi-witness digital editions created using this framework: the diaries of Jacob Bernoulli (1655–1705).^9^ He recorded his reflections in his scientific diary *Meditationes* and documented his journeys in the *Reisbüchlein* travel diary. The edition of *Meditationes* supports historical research, offering multiple transcriptions and translations. The edition of *Reisbüchlein* enhances the travel diary through facsimile–transcription comparison. Both editions demonstrate how modeling at multiple levels (manuscript, entry, page, segment), combined with a user-friendly interface and robust citation system, addresses the five challenges outlined.

As Stäcker (2020) notes, digital editions should be viewed as integrated systems comprising text, metadata, interface, and algorithms, rather than collections of XML files. By leveraging RDF-based multi-level modeling, multi-layered transcriptions, standoff markup, dynamic web presentation, and persistent identifiers, SDEs can overcome the above-mentioned challenges and enhance their scholarly value. Sections  2–4 of the paper detail structural modeling, infrastructure for interactive presentation, and mechanisms for persistent citation, respectively. Beyond efficiency and interoperability, the methodological motivation for modeling diary manuscripts as a region-based knowledge graph is epistemological: it changes what an edition can claim and how those claims can be scrutinized. Traditional editorial workflows often compress interpretive steps, segmentation, reading, normalization, linking, and commentary into a final textual surface (Cugliana et al., 2024). In the proposed framework, editorial assertions are instead externalized as addressable graph statements that remain explicitly connected to the material evidence (facsimile regions) and to the responsible actors (contributors, timestamps, and version histories). This shifts the edition from a static product to a transparent research object whose knowledge claims can be inspected at the level at which they are made: a paragraph, a marginal note, an abbreviation expansion, or a named-entity link. In other words, RDF is not adopted merely to “store” editions, but to represent editorial interpretation as structured, queryable, and citable evidence.

## Representation in RDF

A scholarly edition is more than a reproduction of a historical or literary text: it ensures textual accuracy, provides contextual interpretation, and documents editorial decisions. In RDF-based digital editions, such as those of historical diaries, this involves modeling not only the manuscript and its structural components but also multiple editorial layers, including transcriptions, translations, and commentary. The aim is to create a machine-readable knowledge graph that encodes semantic meaning, enabling reasoning, inference, and efficient querying.

This process relies on ontologies, which define the structured hierarchy of concepts within a domain and describe their essential properties and relationships (Decker & Sergey, 2000). Ontologies formalize knowledge, allowing RDF statements (triples in subject–predicate–object form) to be used in building data graphs that align with the ontology. Together, the ontology graph and data graph form the edition’s knowledge graph. RDF and RDF Schema (RDFS)^10^ provide the basic modeling framework, while *Web Ontology Language* (OWL)^11^ supports richer semantic constructs, logical constraints, and project-specific rules (e.g., a person having only one birthplace). Such constraints can be used to verify consistency across the ontology and data graphs, reasoning, and inference of implicit information. Once defined, knowledge graphs can be queried via SPARQL,^12^ enabling data retrieval and advanced semantic search.

In the following, I outline the ontological constructs, RDF classes, and predicates that must be considered when building multi-layered, region-based scholarly digital editions of historical manuscripts, such as the *Reisbüchlein*. To promote interoperability across RDF repositories and Linked Open Data (LOD), established ontologies are reused, including schema,^13^ FOAF,^14^ the Wikidata ontology,^15^ and the DBpedia ontology,^16^ as well as the Bernoulli-Euler Online (*BEOL*) ontology,^17^ with the prefix beol, which provides constructs for representing historical manuscripts.

A natural point of reference for any ontology-based approach to cultural heritage is CIDOC CRM, which has become a widely adopted standard for modeling artefacts, actors, places, and events in GLAM contexts. CIDOC CRM excels at representing high-level relationships between physical objects, historical events, and agents. Still, it is not designed to model the internal structure of texts, region-based facsimile anchoring, or the editorial processes through which transcriptions, normalizations, and interpretations are produced and revised over time.

Accordingly, the proposed framework aligns with CIDOC CRM where conceptual overlap exists, for example, in the representation of manuscripts as physical objects, persons as actors, places as locations, and events such as creation or revision, but introduces additional domain-specific constructs to represent transcription layers, text regions, standoff annotations, and versioned editorial assertions. These constructs address requirements that fall outside the scope of CIDOC CRM, particularly the need to model scholarly reasoning, evidential grounding, and citation at the level of textual segments. From an interoperability perspective, this layered approach allows CIDOC CRM-compatible descriptions to coexist with and be linked to fine-grained editorial knowledge graphs, ensuring semantic compatibility while preserving the expressive power required for region-based scholarly digital editions. The proposed ontology models SDEs at four levels: manuscript, entry, page, and page segment.

### Manuscript-level RDF representation

**Fig. 1:**
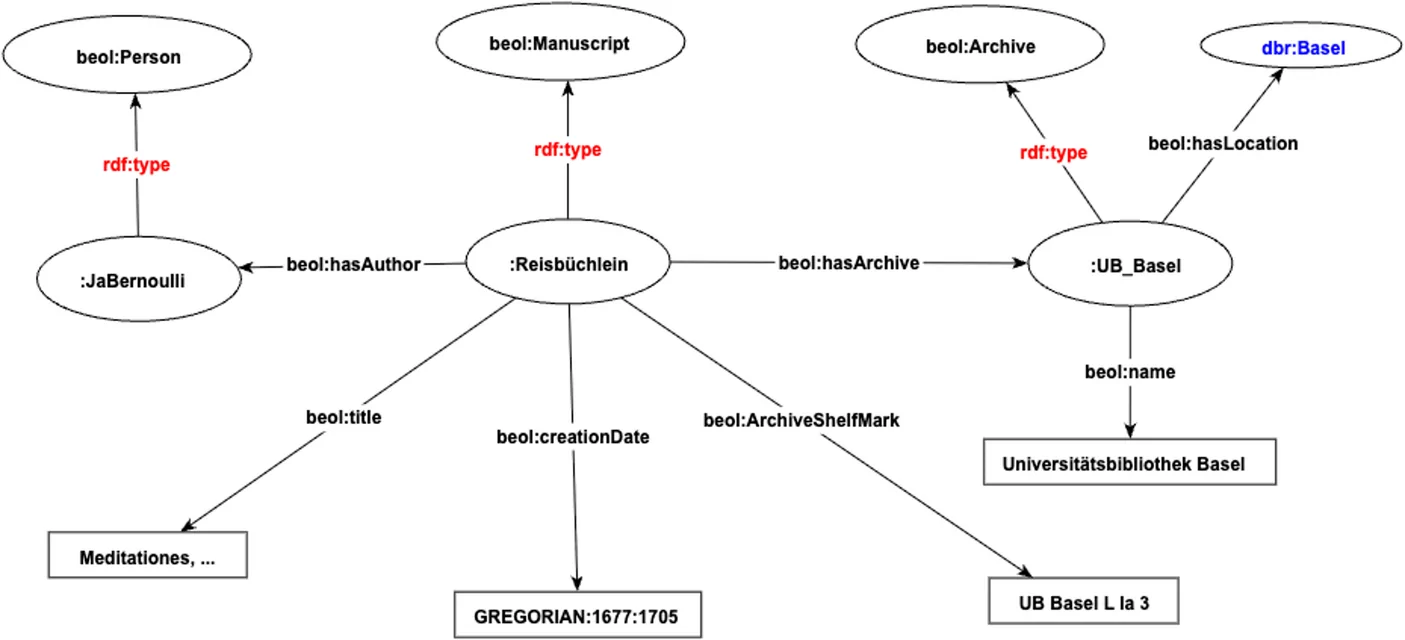
RDF Representation of the *Reisbüchlein* diary (All figures presented in this paper are produced using the yEd graph editor software, see https://www.yworks.com/products/yed.)

A scholarly digital edition of a historical manuscript should provide users with comprehensive information about the artefact itself, including its medium, physical structure, and associated archival data. Accordingly, the first step is to define ontological constructs capable of fully representing the manuscript in all these dimensions. The class beol: Manuscript defines a historical manuscript and is used to represent diaries such as Jacob Bernoulli’s *Meditationes*^18^ and *Resibüchlein*.^19^ Metadata associated with the manuscript is represented through a range of predicates and classes (see Fig. 1).^20^ For example, the beol: hasAuthor represents the manuscript’s author(s), beol: title represents its main title. In this example, the class beol: Archive is used to represent a manuscript’s archival data, including the name and location of the archive, as well as its shelfmark.

Through the predicate beol: creationDate, the manuscript’s creation date can be specified in either xsd: dateTime format or as the project infrastructure’s date value type. The infrastructure employed in this project supports specifying dates with varying levels of precision, including date intervals. Each date can be associated with its corresponding calendar type and era (e.g., BC, CE). To facilitate calendar-independent queries, the system allows the specification of calendar types alongside date values and stores all dates in *Julian Day Number* format (see Alassi & Rosenthaler, 2024, p. 816).

### Entry-level RDF representation

Historical manuscripts can be extensive, often encompassing entire books or collections of writings. Personal diaries are a common example, typically organized into entries that span multiple pages and focus on specific topics, recorded over extended periods of time. For instance, *Meditationes* contains 292 entries reflecting Jacob Bernoulli’s thoughts and analyses on various scientific questions, whereas the *Reisbüchlein* includes 27 entries, each documenting a significant journey or description of a city.

These entries often include titles and the date of composition, spanning multiple pages that contain paragraphs of text, hand-drawn figures, marginal notes, and mathematical formulae. They frequently document a chain of thought that develops across several semantically related entries. In some cases, authors explicitly indicate these connections within the documents, such as through cross-references to other entries or passages. Consequently, the ontology must incorporate constructs capable of representing the interrelated nature of these entries, enabling readers to study the diary both as an integrated whole and at the level of individual pages and entries.

The class beol: ManuscriptEntry represents an individual entry within a manuscript. Each entry may comprise one or more pages and is associated with its parent manuscript via the predicate beol: manuscriptEntryOf. Additional predicates can capture metadata about the entry, including its title, creation date, and topics. For example, the first entry of the *Reisbüchlein*, spanning six pages, records Bernoulli’s journey from Basel to Geneva in 1676.^21^

**Fig. 2:**
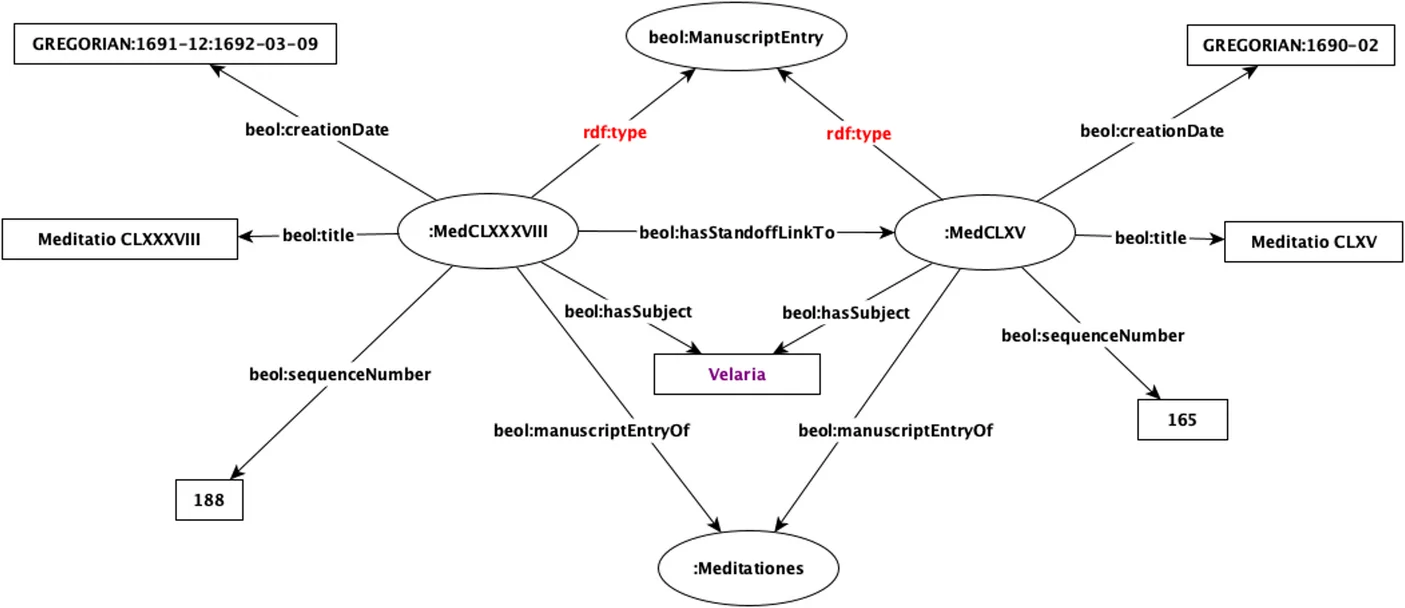
Representation of two thematically linked entries of *Meditationes*

The topic of an entry, represented by the predicate beol: hasSubject, can be identified through topic modeling of the text, derived from subject indices in printed editions (see Alassi & Rosenthaler, 2024, 819–820), or determined through close examination of the content. Multiple entries may share common topics and can be interconnected via internal references. For example, in his detailed discussion of the Velaria problem, Jakob Bernoulli, in entry *Med.CLXXXVIII*,^22^ refers to a passage in *Med.CLXV*,^23^ thereby establishing a semantic link between the two entries. This relationship can be explicitly added to the graph as a standoff link. Figure 2 illustrates the RDF representation of these two *Meditationes* entries using the defined ontological constructs.

### Page-level RDF representation

Each manuscript entry can comprise one or more pages. A multi-witness edition typically presents the witnesses, facsimiles of the pages, and their transcriptions side by side, or in a format that allows readers to compare differences and similarities. This arrangement enables close examination of both the transcriptions and the original documents, allowing verification of transcription accuracy while also providing additional insights from the ink colors, handwriting, and formatting of the originals. Consequently, the edition should include a digitized image for each manuscript page. In LOD-based editions, this can be achieved by storing the image metadata in the knowledge graph, while the images themselves are hosted on an image server. For the *Meditationes* and *Reisbüchlein* editions, the open-source *Sipi*^24^ IIIF^25^-compliant image server is used to store and provide access to facsimile images.

**Fig. 3:**
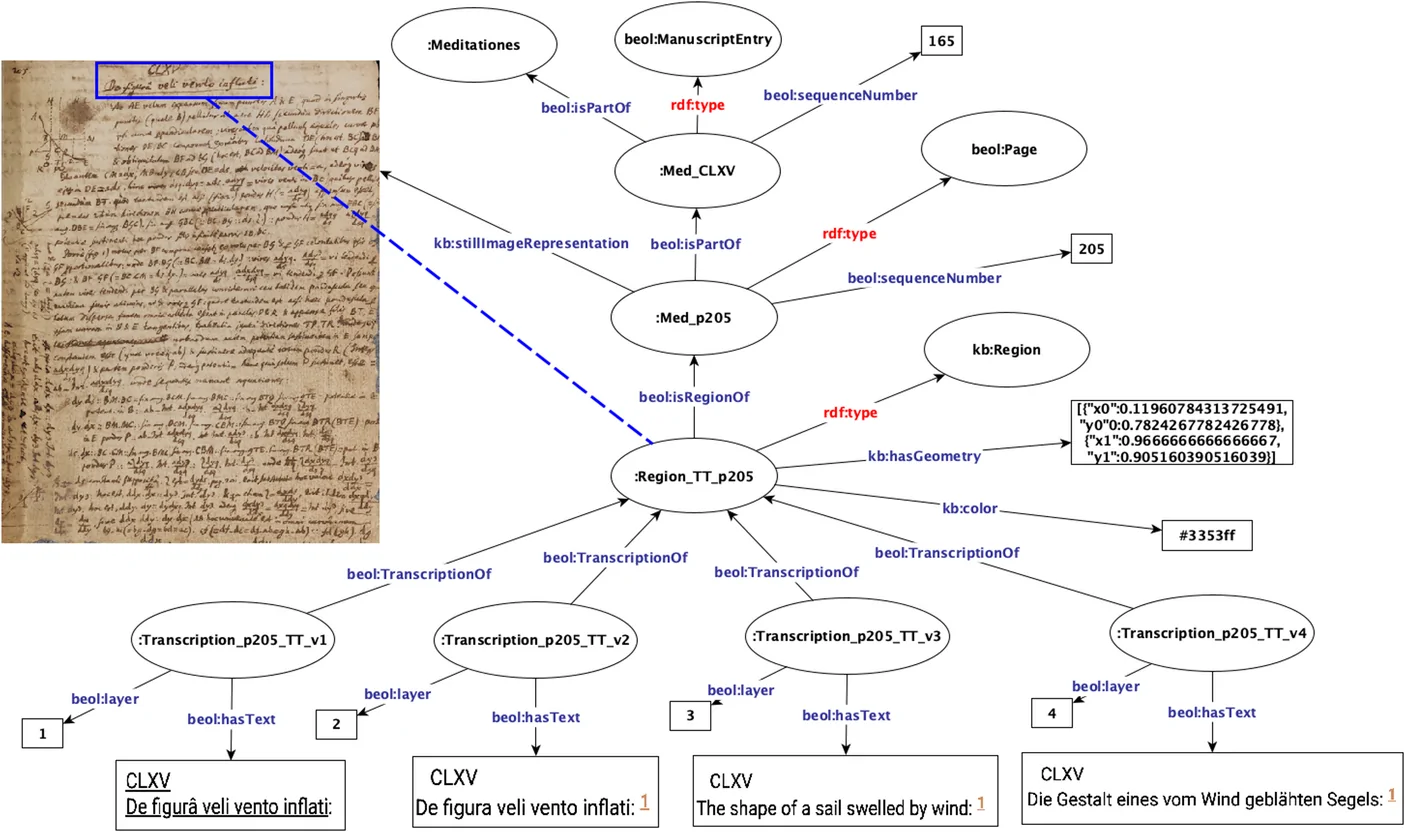
Representation of a region and its transcription layers

As previously mentioned, metadata about a document page should be included in a digital edition but stored separately from the transcriptions to ensure interoperability and facilitate efficient retrieval. In LOD-based editions, it is straightforward to represent a document page along with all its metadata, including sequence number, parent entry, and digital facsimile information, using an RDF class such as beol: Page, as illustrated in Fig. 3. This approach enables the page’s metadata to be stored separately from its transcription, allowing for independent access and querying.

SDEs often provide one or more variations of a document, such as diplomatic, critical, or normalized editions, and may also include translations. As mentioned earlier, to avoid complications from embedded renditions, it is preferable to store different types of transcriptions and translations in separate layers, facilitating accurate parsing and presentation. The digital edition of the *Meditationes* provides five transcription layers per manuscript page: three edition layers (diplomatic, critical, and normalized) and two translation layers (English and German), with the original text in Latin (Alassi, 2020). In contrast, the digital edition of the *Reisbüchlein* offers only a diplomatic transcription layer of the original German text.

The transcription layers are stored individually and linked to the graph node representing the corresponding manuscript page. This setup allows scholars to access and study each transcription independently while consulting the facsimile of the page. In the *Reisbüchlein* edition, the digitized page and its transcription are displayed side by side, preserving the visual structure of the page content within the transcription. The *Meditationes* edition allows readers to toggle between the facsimile and multiple transcription layers, editions, and translations via navigation bars. For both diaries, each manuscript page and its transcription layers are individually citable, see Sect.  4.

A manuscript page typically contains multiple segments, including titles, marginal notes, diagrams, mathematical formulas, and text paragraphs. Consequently, a page’s transcription involves transcribing each of these segments and aligning them precisely to preserve the page’s visual structure. In the following, I outline the segment-level representation of page content and its significance for supporting historical research.

### Segment-level RDF representation

A region-based transcription connects transcribed text to specific spatial areas or segments of a manuscript page, rather than presenting it as a continuous linear stream of text. This approach is especially valuable in scholarly digital editions because it preserves the visual and structural layout of the original document. In many historical texts, spatial arrangement carries meaning: glosses, marginal notes, and interlinear comments often relate interpretively to the main text. By anchoring each textual element to its exact position on the page, region-based transcription preserves these relationships. It maintains the integrity of how the text was originally composed and read.

Each page is composed of multiple region types based on the manuscript content. In the editions of the two diaries discussed here, five types of regions were identified: title, text (philological content), margin (marginal notes), diagrams, and mathematical notations. Each region is annotated on the facsimile using rectangular bounding boxes, created through a combination of image processing algorithms and expert editorial input, and subsequently redrawn as vector graphics (Schweizer et al., 2019). For this purpose, the region annotator tool of the DSP-APP^26^ was employed, which represents each region as an RDF resource of type kb: Region (kb is the prefix for the *knora-base* ontology).^27^ The region’s metadata, including color and coordinates, is stored in the knowledge graph. The bounding box’s upper-left and lower-right corner coordinates are captured via the predicate kb: hasGeometry, while the color code is stored using the predicate kb: hasColor. These properties enable dynamic highlighting of regions on the facsimile during presentation. Each bounding box is color-coded according to its type: red for mathematical notations and diagrams, blue for title regions, purple for marginal regions, and green for text regions.

Once the regions have been identified, their transcriptions can be generated. As noted earlier, many SDEs produce a single transcription document containing multiple transcription types (e.g., diplomatic, critical, normalized), typically indicated through markup tags. The slow processing of these tags during presentation and their inconsistent use across transcriptions are the main reasons that Schmid (2014) recommends storing and presenting transcription layers separately. Following this recommendation, the transcription layers for each page region are created and stored individually in the knowledge graph, represented using the beol: Transcription class. These transcriptions are then linked to the corresponding regions. Figure 3 illustrates the title region^28^ on page 205 of *Meditationes*, belonging to entry Med.CLXV,^29^ along with its critical, normalized, English, and German transcription layers.

As noted earlier, to address the problem of overlapping markup common in TEI/XML editions and to enhance the interoperability of the SDE with machine-learning algorithms for text processing, markup should be stored separately from the transcription texts. This separation allows markup to be modified independently, without altering the underlying transcriptions. Accordingly, in the editions described here, transcription texts are stored as plain text, while standoff markup is maintained separately in the knowledge graph.^30^ The plain text, stripped of markup, can be readily processed by various algorithms, such as NLP and text analysis tools, thereby enhancing the interoperability of the data repository with third-party tools and applications.

However, because a mathematician authored the diaries in this project, they contain substantial mathematical notation, particularly in the *Meditationes* entries. To accommodate this, XML serializations of the transcriptions are also created, incorporating elements with mathematical content in LaTeX, which can be seamlessly rendered for web presentation using MathJax.^31^ These mathematical markups are likewise stored as standoff annotations in the knowledge graph, allowing for overlapping and nesting of mathematical notations, as well as enabling queries for equations based on their standoff markup.

Renear (2004) distinguishes descriptive markups (e.g., < abbr>, < entity> ) from procedural ones (e.g., < i>, < b> ). The project’s ontology includes constructs for representing both descriptive and procedural markup tags, such as internal and external links, as well as formatting elements. Each markup instance is modeled as an RDF resource with properties that define its position in the text using character index ranges. These positional properties are crucial for accurately rendering the markup in the web presentation of transcriptions and also enable targeted queries in the knowledge graph for annotations with specific types of standoff markup. For example, one could query for passages where a particular person is mentioned or retrieve all entries in which Bernoulli wrote a specific phrase in italics.

**Fig. 4:**
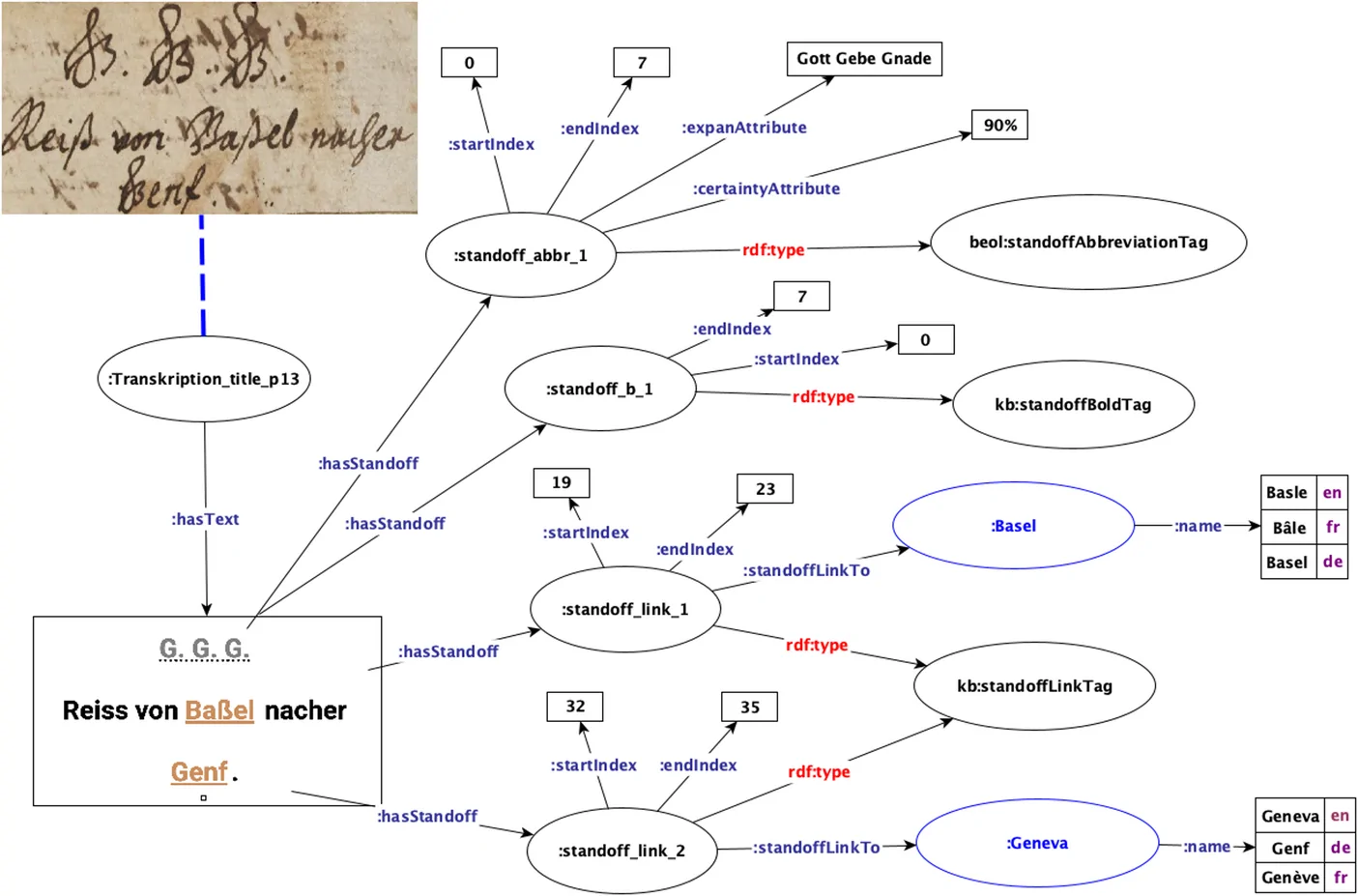
Representation of a text with standoff markup in the knowledge graph

Figure 4 shows the transcription of the title region^32^ from page 13^33^ of *Reisbüchlein*, along with three associated markup annotations: references to the cities Basel and Geneva, and the acronym *G. G. G.* presented in emphasized form. These annotations and transcriptions are created either manually by an editor or automatically via *Transkribus*^34^ or the self-developed NLP pipeline *textToRDFGraph*.^35^ The level of certainty, whether from the editor or the algorithm, regarding the meaning or accuracy of an annotation can be recorded in the knowledge graph using the: certaintyAttribute property. For instance, the collaborating editor of *Reisbüchlein* was 90% certain that the abbreviation *G. G. G.* stands for *Gott Gebe Genade*. Storing the level of certainty allows researchers to assess the reliability of annotations, make informed decisions about their use, and prioritize verification efforts where confidence is lower.

This certainty information can then be reflected in the presentation of the text, e.g., with gray font color. This accurate representation not only makes the edition more trustworthy for the reader, but it also allows the reader to filter out all annotations with accuracy above a certain threshold, e.g., 85% or higher (Ammann & Alassi, 2024). Similarly, the confidence degree of the algorithms in producing the result can also be added to the knowledge graph. For example, the model used for the named entity recognition process had identified the geopolitical entities *Basel* and *Genf* (i.e., *Geneva*) with certainty close to 100%. This could also be added to the markup resources.

Therefore, the abbreviation is stored in the knowledge graph as an instance of the standoff abbreviation markup, together with its position in the text, its expanded form, and the editor’s confidence level in this interpretation. Since the acronym is emphasized in the text, it is also associated with a nested bold markup instance, stored separately as part of the standoff bold markup class. Furthermore, the two cities referenced within the text are represented as RDF resources: Basel and: Geneva, of type beol: Location. Edges are added to the graph to link the transcription to standoff link markup resources corresponding to these locations.

Upon presentation on the web application, the standoff markups are converted to HTML tags, and transcriptions to HTML documents. For example, the abbreviation is then represented as.

< abbr expan="Gott Gebe Gnade” certainty="90%”>< b > G. G. G. </b></abbr> with nested bold markup (< b> ) in HTML (< strong> ). This enables the presentation of the expanded form in a pop-up box, and the uncertainty in the annotation is reflected in the presentation through the use of gray font color (see Fig. 6). References to graph resources (locations, persons, etc.) are replaced with hyperlinks to the corresponding resources in the graph.

Since the IRI of the target resource is used to create hyperlinks, the graph nodes representing text values are directly connected to the nodes representing the corresponding entities, just like any other relationship in the graph. This structure enables queries for texts based on their references; for example, one could retrieve all text regions in which Bernoulli mentions the city of Geneva. Such a query searches the knowledge graph for transcriptions that have a standoff link to a resource whose name includes “Genava.” Because the ontology supports multiple names with different language tags for each entity, the query would also return texts containing “Geneva,” “Genf,” or “Genève” from anywhere in the project’s knowledge graph. In this way, the project’s approach enables language-independent queries across the manuscripts’ content (Alassi, 2023).

**Fig. 5:**
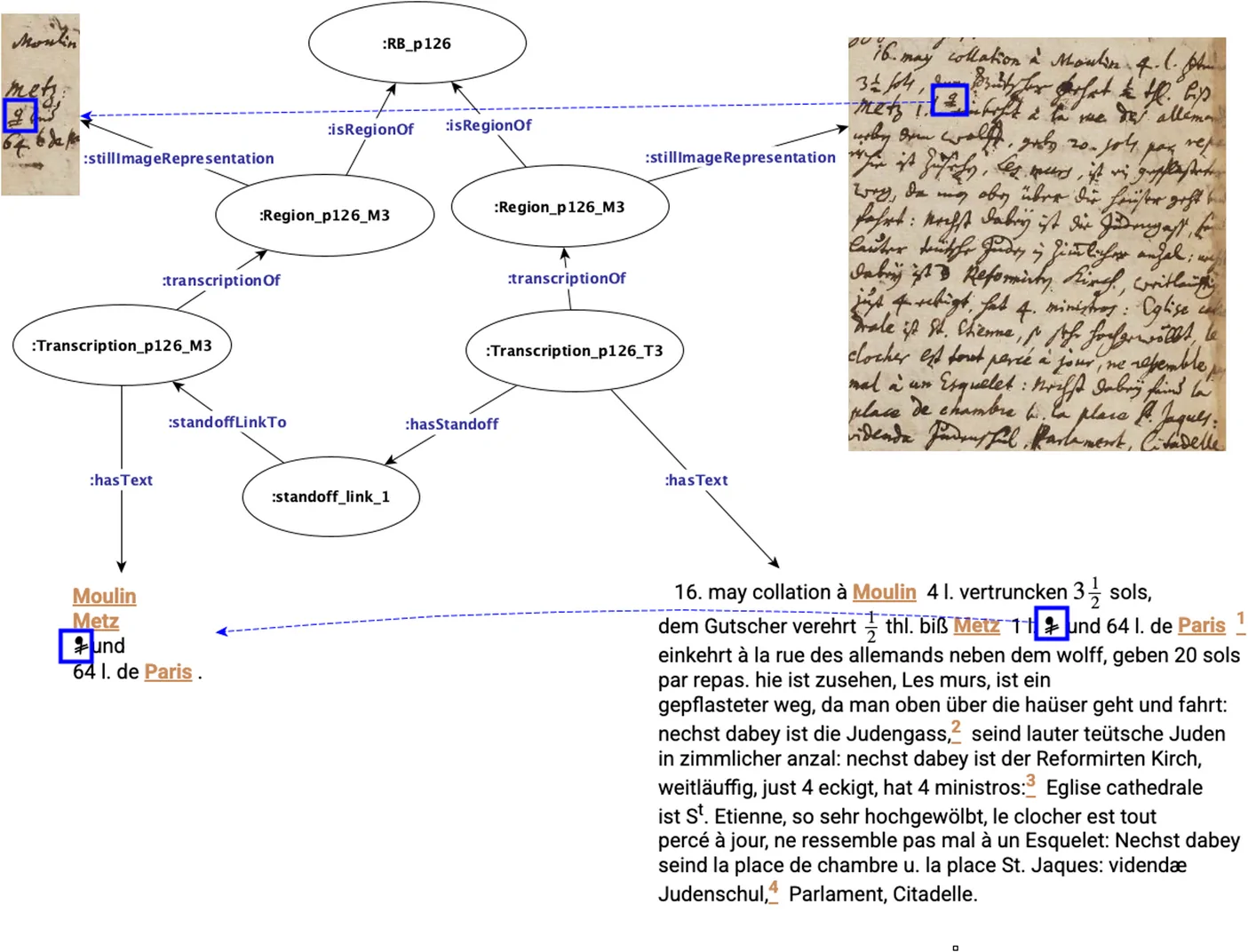
Representation of inter-regional references within page content

Text passages can also contain references to other pages or passages within the same manuscript. These inter-regional references connect sections that form part of the same thematic discussion, often appearing in margins or on separate pages as afterthoughts. Capturing these linkages in the knowledge graph enables coherent navigation between thematically related passages. Figure 5 illustrates this with a marginal note^36^ on *Reisbüchlein’s* page 126,^37^ which belongs to the 18th entry^38^ describing Bernoulli’s journey from Paris to Basel. The marginal region and its normalized transcription reference several cities, as well as another text region on the same page. This reflects Jakob Bernoulli’s internal referencing system: a symbol in the margin links the note to a specific paragraph in the main body of the page. Consequently, a query for all regions linked to the third paragraph^39^ of *Reisbüchlein’s* page 126^40^ would return the marginal region: Region_p126_m_3 together with its transcription.

A scholarly digital edition is typically research-driven, presenting not only a curated version of a text but also the results of in-depth academic analysis of its thematic content. For example, the digital editions of the two diaries include research-based commentaries enriched with extensive references to both primary sources and secondary literature. These commentaries, produced through meticulous examination of individual entries, are stored separately from the transcriptions using the beol: Comment class.

The text of these annotations can be enriched with internal and external links, enabling editors to clarify obscure references, provide historical context, or document editorial interventions such as corrections, conjectures, or emendations. To ensure the data remains authoritative and citable, provenance information, such as the contributing editor and the date of annotation creation, is preserved in the knowledge graph. Editorial comments are linked to the transcriptions and presented alongside the relevant transcription segments, using superscript numbers similar to traditional footnotes (see Fig. 5).

The knowledge graph containing the SDE data is built upon the ontologies described above and can be stored in any RDF triplestore, making it accessible through the triplestore’s SPARQL endpoint. However, to ensure effective access and exploration, an infrastructure is needed that provides RESTful APIs for retrieving, querying, and modifying the data, if necessary. Additionally, a dedicated web application is crucial for presenting the SDE in a functional and user-friendly manner. The following section outlines the infrastructure and web application developed to present the historical diary SDEs in an interactive form, enabling users to perform sophisticated queries and study the content efficiently.

## Interactive presentation on the web application

Web applications for digital editions provide dynamic, accessible platforms for editing, presenting, and interacting with historical texts. Unlike static print editions, these digital environments support rich features such as multi-version views, integrated annotations, manuscript facsimiles, and interactive tools for navigation and search. By leveraging technologies such as TEI/XML, IIIF, Linked Open Data, and visualization tools, web applications enable scholars to link transcriptions with images, provide variant readings, and contextualize texts within broader historical or linguistic frameworks. They also allow collaborative editing, user-driven commentary, and segment-level referencing, making them valuable not only for academic research but also for teaching and public engagement with cultural heritage.

In LOD-based digital editions, the knowledge graphs stored in a triplestore can be made openly accessible through the triplestore’s SPARQL endpoint. Requests for data can be made through this endpoint by submitting SPARQL queries in the payload of HTTP requests, and responses can be retrieved in formats such as JSON-LD or RDF/XML. Through SPARQL SELECT queries, users can retrieve RDF triples matching the search criteria, and through SPARQL CONSTRUCT queries, a subgraph can be retrieved. However, making the SPARQL endpoint of a triplestore openly accessible can pose significant risks. Unrestricted access may lead to performance issues, as poorly constructed or malicious queries can overwhelm the server and degrade availability. It also raises security and data integrity concerns, especially if sensitive or unpublished data is included. Furthermore, open endpoints can expose internal data structures or ontologies not intended for public use, which may lead to misuse or misinterpretation. To mitigate these risks, it’s advisable to apply access controls, rate limiting, or offer RESTful APIs for a controlled public interaction instead.

**Fig. 6:**
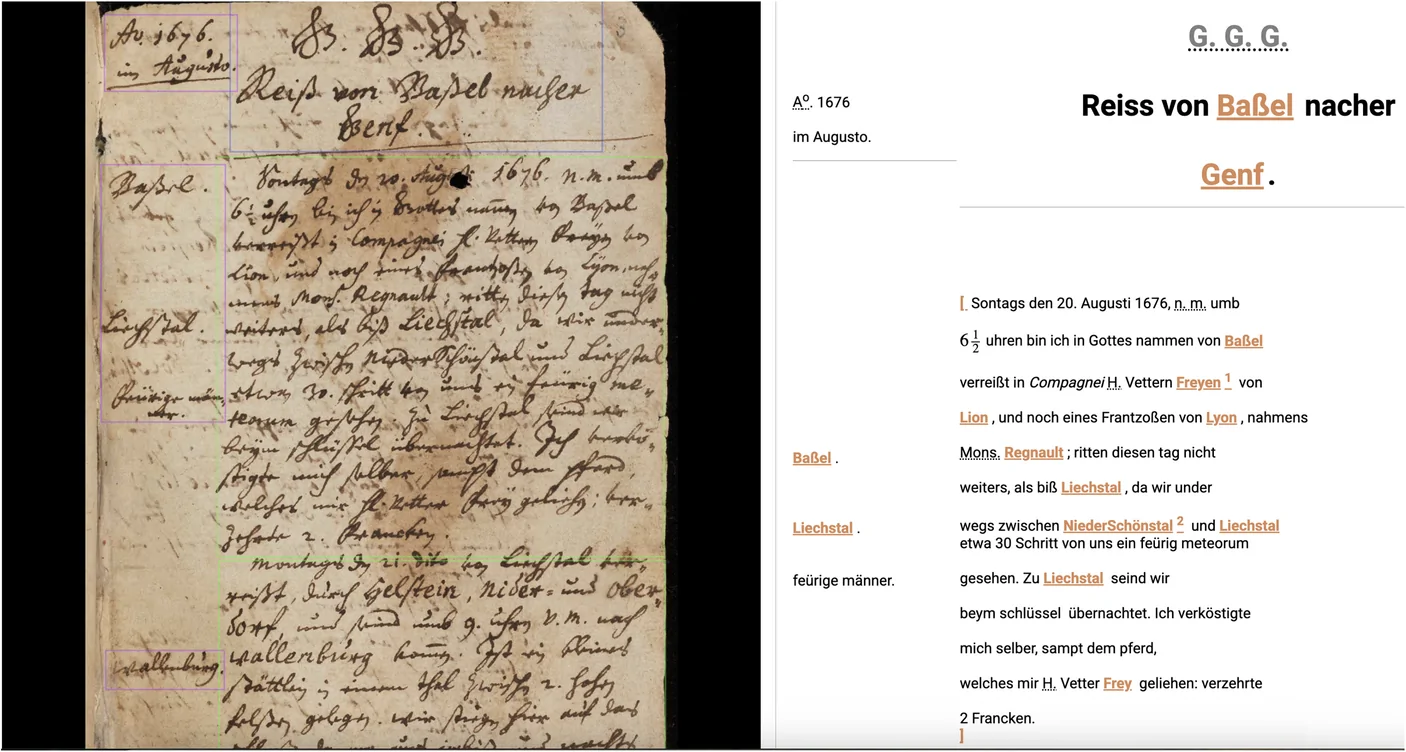
Web representation of page 13 of *Reisbüchlein* and its transcription

The API, used for the suggested framework, supports permission control, for instance, by granting read-only access to unauthenticated users while allowing project members to edit content. Data owners can choose to make the entire graph openly accessible or restrict parts of it to members and administrators. In this way, the API can verify a user’s authorization before granting access to graph data and retrieve information from the triplestore according to their access rights. By integrating query validation, the API can also pre-screen SPARQL queries to ensure they are well-formed and syntactically correct, reducing the risk of execution errors or misuse. Once query results are returned from the triplestore, the API can restructure and format them for seamless integration into web applications and browser rendering, such as in HTML or JSON. Moreover, APIs can facilitate access to resources stored across different servers; for example, Fig. 6 illustrates how the API retrieves both RDF data from the triplestore and a facsimile segment from the image server in response to a request for a specific page region.

For the editions described in this paper, we rely on the infrastructure provided by DaSCH,^41^ which ensures the long-term preservation and open access of humanities data in Switzerland. This infrastructure includes an RDF triplestore (DSP-DB) for storing the editions’ knowledge graphs, an open-source RESTful API (DSP-API)^42^ for authorized access to the graph, and an open-source IIIF-compliant image server (Sipi) for storing and retrieving facsimiles and their segments via IIIF URIs and manifests. It also provides a generic web application (DSP-APP),^43^ based on the DSP-API, that presents project data graphs and ontologies in a user-friendly interface. The application offers query-based graph analysis tools, whose web components reflect the project’s ontologies, thereby eliminating the need for users to master SPARQL or comprehend ontology details. Based on the search criteria entered, the application automatically generates the underlying SPARQL query, while also offering advanced query tools for expert users to enter their own SPARQL statements with greater complexity. Furthermore, DSP-APP fully supports CRUD (Create, Read, Update, Delete) operations, enabling project members to edit data and ontology graphs, while restricting anonymous users to read-only access.

Although this type of generic web application is well-suited for presenting data from various projects, it does not address the specific needs of individual projects. Depending on the nature and characteristics of the historical manuscripts, SDE projects may employ different strategies for presenting the edition online. Some editions, such as the *Briefportal Leibniz*,^44^ provide only a single primary transcription. In contrast, others, such as *The Newton Project*,^45^ offer multiple transcription types (e.g., diplomatic and normalized) accompanied by external links to facsimile archives. Still others, such as *HallerNet*,^46^ present facsimiles and their corresponding transcriptions side by side, facilitating direct visual-textual comparison.

In general, edition projects require web applications that offer facsimiles, manuscript data, transcripts, translations, and other resources in a manner that reflects the complexity of the data, yet keeps the presentation simple enough to allow in-depth study of the texts. The project-specific web applications can then contain user guidelines, a description of the project and its contributors, as well as query-based research tools that facilitate the analysis of editions, visualizations, and navigational components tailored to a project’s objectives, presenting a historical document and its corresponding edition.

The digital editions of Jacob Bernoulli’s *Meditationes* and *Reisbüchlein* diaries, as discussed in this paper, are both integrated into the *BEOL* web application.^47^ This platform presents the editions in an interactive, multi-layered, and region-based format, providing scholarly introductions to each historical diary and a complete list of their entries. Comparable to the DSP-APP, the BEOL application incorporates advanced search tools that support the exploration of the editions’ underlying knowledge graphs and enable the retrieval of graph data pertinent to specific research inquiries. Given the distinct nature of the two works, *Meditationes*, a scientific diary containing complex mathematical diagrams and extensive formulae distributed across multiple pages with layered transcriptions and translations, is presented in a manner tailored to its scientific and technical content. In contrast, the *Reisbüchlein* travel diary is presented in a format that suits its narrative and geographical focus.

### Entry-level web presentation

The entries in *Meditationes* often comprise multiple pages devoted to a single topic, with Bernoulli frequently presenting mathematical analyses that extend across several consecutive pages. To support coherent, uninterrupted study of these extended arguments, and to avoid page breakages within mathematical notations, the transcriptions of an entry’s pages are compiled and displayed as a continuous text. For each entry, the available transcription and translation layers are listed, enabling users to select or switch between them as needed. Accompanying metadata, including the entry’s title, links to the original manuscript, and the name(s) of the editor(s), is provided alongside a citation link, as detailed in Sect.  4. From any transcription layer, users can navigate directly to the individual pages of the entry and access their corresponding facsimiles.

Each entry in the *Reisbüchlein* documents either a specific journey or a city, often spanning multiple pages on which Bernoulli meticulously recorded the stages of travel. These accounts include details such as departure and arrival dates, accommodations, modes of transportation, travel companions, and associated costs. For each entry, the application presents comprehensive metadata, including the title, the sequential number of the entry within the diary, links to the corresponding *Reisbüchlein* manuscript, and a citation link for the entire entry. For journey-related entries, the application additionally provides a map visualization of the route alongside tabular representations summarizing the overall journey and its individual stages. These visual and tabular elements offer readers a synthesized overview of the trip, serving as an entry point for a more detailed examination of the text.

Since Bernoulli’s descriptions of his journeys often included drawings of symbols and logos he encountered, as well as the names of people he met, frequently with incorrect spellings, I opted for a side-by-side presentation of each page’s facsimile and transcription. This format allows users to directly compare the edited text with the original manuscript, facilitating verification, interpretation, and a deeper understanding of Bernoulli’s notes. From an entry, users can access its complete transcription, which is guided by the application, and view the representation of the entry’s first page. Navigational bars enable users to turn the pages, allowing them to explore the diary’s content sequentially and study the manuscript in a manner closely aligned with the original reading experience.

### Page-level web presentation

Upon accessing a page through the application, the API utilizes the page’s unique identifier (IRI) to retrieve the corresponding RDF resource from the knowledge graph by issuing SPARQL queries to the triplestore. From the retrieved resource data, the API obtains the IIIF-URL of the page and uses it to fetch the facsimile from the image server. The application subsequently presents the properties of the page resource, along with its citation link, enabling scholars to reference a specific page of the manuscript directly and accurately.

The application then retrieves the annotations of all regions associated with the page from the knowledge graph. Using the geometry and bounding box information of each region, it overlays the regions onto the page facsimile (see Fig. 6 for an example of the page representation). To facilitate a detailed study of specific passages, the regions on the facsimile are made interactive. Each facsimile segment is linked to its corresponding region representation via its IRI. When a user clicks on a region, the application navigates to the representation of that region along with its image segment and transcription, as described in Sect.  3.3.

Subsequently, the application retrieves the transcriptions of all regions associated with the page using their IRIs. These transcriptions are then aligned to construct a coherent and comprehensive representation of the entire page, preserving the structure of titles, marginal notes, and paragraphs as accurately as possible. To achieve this, an XSLT transformation ruleset generates an HTML table with centralized cells for titles, a dedicated column for marginal regions, and a column for main text paragraphs. Each row of the table corresponds to a paragraph and its associated marginal region. The beginning and end of each region’s transcription are marked with brackets linked to the region’s representation, Fig. 6, enabling scholars to navigate seamlessly from a page-level view to the representation of a specific segment for detailed study of the region and its transcription or to access the segment’s citation link.

### Segment-level web presentation

As noted above, segment-level representations of historical texts enable precise citation, targeted annotation, and the tracking of textual variants at the level of individual paragraphs or other meaningful units, such as marginal notes. This granular structure allows scholars to reference and compare specific text regions across different editions, languages, or manuscript witnesses. Moreover, it facilitates the integration of external data, such as editorial commentary, with discrete textual units, thereby enhancing interoperability and promoting reuse. If various contributors had created transcriptions of regions, accurate contribution details are added to each region and displayed to the users. A region-based approach is therefore fundamental for creating interactive, modular, and richly annotated digital scholarly editions.

When a user selects a region of historical text, either by clicking on its facsimile segment or transcription delimiters, the application retrieves the corresponding region resource from the knowledge graph via the API. In addition to the region’s metadata properties, the API also obtains its image segment along with the associated transcription(s) and markup data. Figure 6 illustrates the workflow for retrieving both the image segment and the RDF data for a region from the underlying storage systems and displaying them in the project’s web application.

**Fig. 7:**
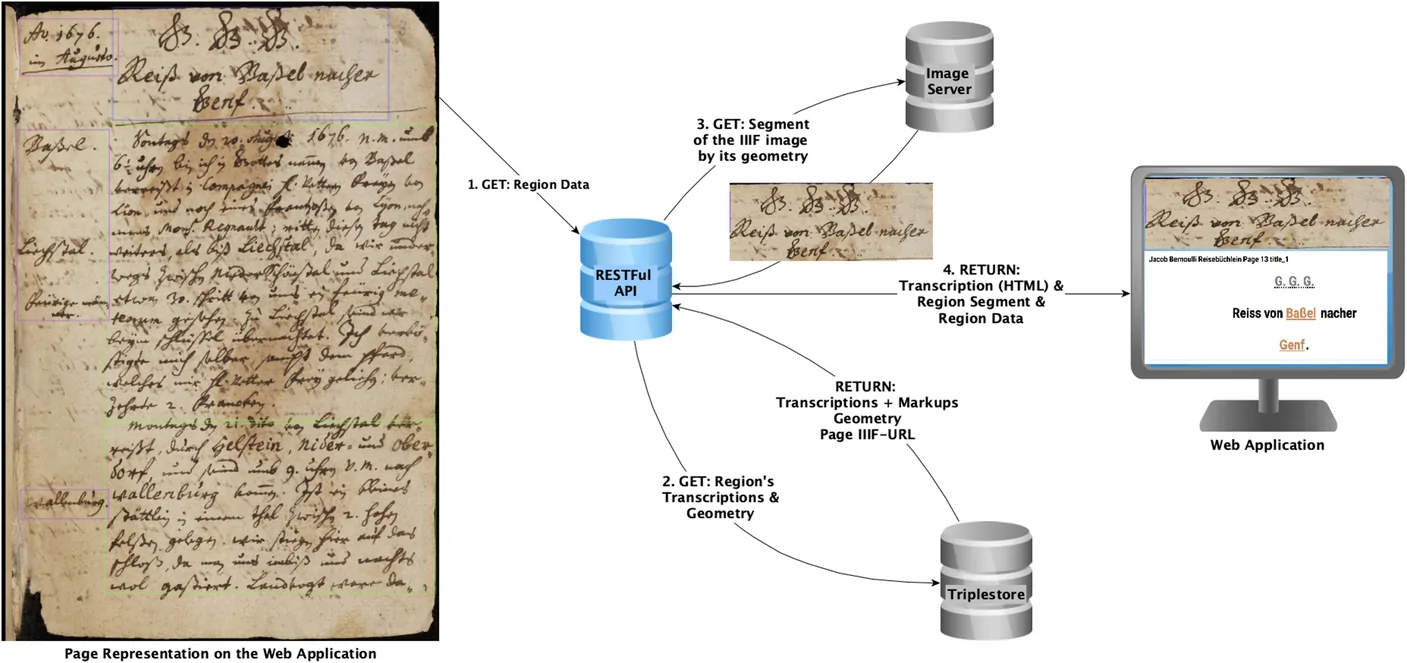
Workflow of the interactive region retrieval

As illustrated in Fig. 7, when a facsimile or transcription segment is selected, the web application issues an HTTP request to the API using the region’s IRI to retrieve its corresponding data. The API executes this process in three steps. First, it retrieves the RDF resource representing the region via its IRI by issuing a SPARQL query to the triplestore, which returns the resource along with all associated properties, including the region’s geometry, available facsimiles, and transcriptions. Second, using the geometry information from the triplestore’s response and the page’s IIIF-URL, the API retrieves the image segment from the image server. Finally, the API combines the region’s transcriptions with their associated markup to generate HTML representations of the edition layers (see Alassi & Rosenthal, 2024, Fig. 9). Links to relevant comment resources are also incorporated into the transcriptions when available. The API then returns the region’s HTML transcription, its image segment, and additional data to the web application.

The web application visually highlights the selected segment on the page’s facsimile and presents the properties of the corresponding region resource, including its transcription and links to relevant editorial comments. For each segment, the application also provides a citation link, enabling scholars to reference a specific segment precisely in their research outputs.

## Citation and referencing

Citing and referencing are vital for scholarly transparency; however, digital environments pose challenges such as broken links and repository migrations, which threaten the long-term reliability of citations. Traditional citation methods, developed for print, often fail to guarantee persistent access to SDEs. To address this, Bleier (2021) advocates the use of stable identifiers (e.g., DOIs, URNs, PIDs, ISBN-A) and fragment identifiers for precise citation. Without them, digital editions risk becoming uncitable.

The paper presents a method for citing SDE components, ranging from entire manuscripts and pages to specific text segments, using identifiers from RDF knowledge graphs (IRIs). While IRIs denote identity within a graph, they are not inherently resolvable web addresses, limiting their effectiveness. Instead, the project implements *Archival Resource Key* (ARK) URLs, persistent identifiers designed to remain reliable regardless of system changes (Kunze, 2019). ARKs support metadata, provenance, and qualifiers such as segment or version references, making them particularly suitable for RDF resources in linked data contexts.

An ARK URL encodes repository and resource details and may include timestamps to reference specific versions. Within the SDE infrastructure, ARK resolvers translate ARK URLs into IRIs and direct users to the corresponding web presentation. All entities in the knowledge graph, regions, pages, persons, transcriptions, etc., can thus be cited precisely. For example, a marginal region of the *Reisbüchlein* can be cited with an ARK link that reliably resolves to its presentation in the web application, ensuring accurate and reproducible reference.

This system also addresses URL corruption during repository or server migrations. ARK-based citation links remain valid, as only the resolver needs to be updated to redirect to the new infrastructure. Citation links can be either pre-generated and stored in the knowledge graph or created dynamically upon request. Projects that maintain version histories, such as BEOL, can include timestamps in ARK URLs, ensuring that citations resolve to the exact state of a resource at the time of citation. A further challenge is the dynamic nature of digital objects. While corrections and updates improve SDE quality, they risk undermining citation stability: the version cited by a scholar may later differ from the version readers access. Unless a versioning system is in place, citation links typically resolve to the most recent version. The infrastructure described here addresses this by incorporating RDF-based versioning that maintains a history of changes to the RDF resources and object values, enabling scholars to cite specific versions of resources and ensuring long-term reliability and consistency. 48

Combining ARK-based persistent identifiers with RDF knowledge graphs and versioning creates a robust framework for citing digital scholarly editions. This system ensures precise, durable, and reproducible references, overcoming the instability of conventional URLs and supporting the long-term accessibility of digital scholarship. The editions described in this paper are integrated into DaSCH, the Swiss national infrastructure for humanities data preservation, ensuring the long-term availability of the editions’ knowledge graphs and the reliable resolution of ARK URLs to their corresponding resources (Alassi & Rosenthaler, 2024, p. 832).

## Discussion

While the preceding sections have described the technical and methodological components of the proposed framework, this discussion turns to a more fundamental question: how and why these modeling choices matter for textual scholarship. In particular, it examines the epistemological implications of representing historical manuscripts as multi-granular, region-based knowledge graphs and of treating editorial outputs as structured, inspectable scholarly claims rather than as static textual products.

A first epistemological gain lies in the strengthened evidential status of transcriptions through their anchoring to bounded facsimile regions. In this model, a transcription is not merely a string of characters but an assertion about a specific area of a particular manuscript page, retrievable and citable as such. This reduces the persistent epistemic gap between “what the edition says” and “what the document shows,” because the evidential basis of a reading is embedded directly in the edition’s data model rather than implied by editorial authority or deferred to narrative explanation. Region-level anchoring also allows uncertainty to be localized: ambiguity in an abbreviation expansion, a difficult hand, or a damaged surface can be recorded precisely where it occurs, instead of being absorbed into an undifferentiated confidence in “the transcription” as a whole.

A second consequence concerns interpretation and authority. By modeling multiple editorial layers and transcription versions as separate, addressable resources associated with the same region, the framework operationalizes the idea that editorial outputs are contingent hypotheses shaped by method, purpose, and scholarly perspective. Diplomatic, critical, normalized, and translated versions can coexist without being hierarchically subordinated to a single “definitive” text. Because each layer and version carries its own identifier, provenance, and timestamp, readers can retrieve and cite the specific interpretive state that supports their argument, rather than being forced to reference an evolving and implicitly authoritative “latest view.” Authority thus shifts from a stabilized editorial surface to an explicitly documented space of alternatives in which differences between readings can be analyzed, compared, and reused.

The knowledge-graph structure further renders the editorial process itself analytically accessible. In many editions, revisions and corrections are editorially meaningful but methodologically opaque once a new version replaces an old one. By contrast, RDF-based versioning combined with timestamped ARK citations enables scholars to trace how readings change over time: which segments were revised, by whom, and in response to which kinds of evidence or commentary. The edition thus becomes a record of scholarly reasoning rather than merely of its outcomes, supporting accountability (editorial actions remain attributable), traceability (claims remain linked to evidence), and reproducibility (a cited version can be recovered), all central criteria for scholarly trust in digital environments.

The use of standoff annotation reinforces this epistemological shift. Modeling links, entities, abbreviations, formatting, and commentary as independent resources tied to explicit character offsets prevents scholarship from being forced into a single dominant hierarchy. Competing analytical perspectives, such as layout, semantics, named entities, mathematical notation, or marginalia, can be layered without overwriting one another, while remaining queryable and referable. Interpretation is not eliminated or neutralized; rather, it is made explicit, inspectable, and computationally addressable at the level of discrete scholarly claims. In this sense, the edition functions as a structured argument about the manuscript, in which evidence, reasoning, and responsibility are represented within the same formal model.

This framework can also be situated within the broader debate on so-called “next-generation” markup languages, which has emerged in response to the structural limitations of hierarchical XML models when faced with overlapping, discontinuous, or multi-perspectival textual phenomena (Haentjens-Dekker et al., 2025). Proposals in this debate aim to move beyond document-centric hierarchies toward representations that better reflect the layered, interpretive, and often conflicting structures inherent in historical texts. The standoff/RDF approach adopted here aligns with this trajectory by decoupling transcription from annotation and by modeling interpretive statements, structural features, and semantic links as independent, addressable resources. Rather than attempting to resolve overlap through increasingly complex inline encodings or alternative grammars, the knowledge graph treats overlap as an epistemic condition to be represented explicitly. This allows multiple analytical perspectives, layout, linguistic structure, named entities, editorial commentary, or mathematical notation to coexist without being forced into a single dominant structure. Markup is thereby reframed not as a fixed encoding of textual “truth,” but as a collection of situated scholarly assertions that remain open to revision, comparison, and reuse.

From the perspective of citation and reuse, the proposed framework directly engages with concerns articulated by Cugliana et al. (2024), who argue that next-generation digital editions must move beyond static referencing toward models that expose provenance, authority, and the contingent nature of scholarly assertions. By embedding citation mechanisms at the level of regions, transcription layers, and versions, the knowledge graph allows scholars to cite not only an edited text, but a specific editorial claim together with its evidential basis and responsible contributors. This redistributes authority away from a single, stabilized editorial surface and across explicitly documented acts of transcription, normalization, and interpretation. Different versions of a transcription, produced by different contributors at different times, can coexist and remain citable, making disagreement, revision, and alternative readings visible parts of the scholarly record rather than anomalies to be resolved or suppressed. Citation thus becomes an epistemic operation rather than a purely referential one: it enables scholars to align their arguments with particular interpretive states of an edition and to reuse those states transparently. Authority, in this model, is grounded not in editorial finality but in traceable evidence, accountable processes, and the explicit articulation of scholarly choices.

Recent graph-based approaches to text modeling, such as the *Applied Text as Graph* (ATAG) methodology (Kuczera, 2025), further underscore the broader disciplinary shift toward making editorial structures, addressability, and process explicit in digital editions. While ATAG pursues this goal through character-level modeling, the framework proposed here adopts region-based granularity as a deliberate methodological choice, aligning textual assertions directly with bounded facsimile evidence. This allows transcription layers and annotations to be treated as situated scholarly claims about specific manuscript regions, supporting efficient querying, citation, and reuse at scales that correspond to common hermeneutic practices. At the same time, from an architectural and technical perspective, the framework does not preclude finer granularity: transcriptions can, where required by the research question, also be modeled at the character level rather than as paragraph- or sentence-sized units. By privileging evidential grounding and interpretive accountability over maximal textual atomization, the presented approach emphasizes transparency and scholarly responsibility while providing granular annotations of small text regions. Taken together, these considerations clarify how the proposed framework contributes not merely to a new technical implementation but to a reconfiguration of editorial practice in which evidence, interpretation, authority, and citation are structurally integrated.

## Conclusion

This article has presented a comprehensive approach to building and presenting scholarly digital editions grounded in RDF-based knowledge graphs, with a particular focus on manuscript pages, textual regions, and their facsimiles. By representing each element, from entire manuscripts to granular text regions, as a distinct RDF resource with a unique IRI, the framework enables precise, segment-level citation, targeted annotation, and interoperability across editions, languages, and manuscript witnesses. The web application described here operationalizes this model by dynamically retrieving and presenting pages, regions, transcriptions, and annotations, thereby offering scholars an interactive, modular environment for close reading and research.

As the discussion has shown, the primary contribution of this framework is not merely technical but epistemological. By modeling transcriptions, markup, and commentary as addressable scholarly assertions anchored to specific facsimile regions and enriched with provenance and versioning information, the edition makes the evidential basis of readings explicit and editorial responsibility traceable. Multi-layer and multi-version representations decenter authority from a single edited surface and instead support plural, citable interpretive states that can be compared, reused, and examined. In this sense, the knowledge graph does not simply represent the manuscript; it represents the scholarly reasoning applied to the manuscript, enabling transparency, accountability, and reproducibility in ways that are difficult to achieve with monolithic transcription files or purely presentational web editions.

A key enabling factor of this epistemological shift is the use of persistent, resolvable identifiers. By integrating ARK URLs into the infrastructure, every resource can be cited reliably, even across repository migrations or changes to the web application. Timestamped ARK URLs allow citations to resolve to the exact state of a resource at the time of consultation, thereby supporting reproducibility and long-term scholarly trust. This stability, combined with the underlying RDF graph, also facilitates the linking of internal and external resources, enabling rich metadata integration, commentary, and cross-referencing. Together, these technical and methodological elements form a robust foundation for the creation, presentation, and durable citation of digital scholarly editions.

Looking forward, the availability of a fully structured knowledge graph opens the way for integrating an advanced tool based on *Graph Retrieval-Augmented Generation* (GraphRAG) into the web application. Such a tool would enable scholars to pose research questions in natural language, translate them into structured SPARQL queries, and generate coherent textual responses explicitly grounded in the graph resources that match those queries. By combining the precision of structured knowledge graphs with the interpretive accessibility of generative methods, this approach offers a promising path toward interfaces that are both computationally powerful and epistemically transparent. Developing such GraphRAG-based tools is a goal of an ongoing project building on the framework presented here.

## Data Availability

All project data are available on the DaSCH infrastructure accessible through the following link: https://app.dasch.swiss/project/yTerZGyxjZVqFMNNKXCDPF/description.

## Footnotes

1
https://tei-c.org/.

2
https://www.newtonproject.ox.ac.uk/.

3
https://www.hallernet.org/.

4
https://www.w3.org/TR/rdf11-concepts/.

5 See Hyvönen et al. (2023), https://seco.cs.aalto.fi/projects/rrl/.

6 See Münnich and Ahrend (2021), https://www.dasch.swiss/project/anton-webern-gesamtausgabe.

7
https://weber-gesamtausgabe.de/en/Index.

8 See Alassi and Rosenthaler (2024) and the platform https://bernoulli-euler.dhlab.unibas.ch/.

9
https://bernoulli-euler.dhlab.unibas.ch/biography/Jacob%20I%20Bernoulli.

10 https://www.w3.org/TR/rdf-schema/.

11 https://www.w3.org/TR/owl2-rdf-based-semantics/.

12 https://www.w3.org/TR/sparql11-query/.

13
https://schema.org/docs/datamodel.html.

14
http://xmlns.com/foaf/spec/.

15
https://www.wikidata.org/wiki/Wikidata: WikiProject_Ontology.

16
https://www.dbpedia.org/resources/ontology/.

17
https://app.dasch.swiss/project/yTerZGyxjZVqFMNNKXCDPF/ontology/beol/editor/classes.

18
https://ark.dasch.swiss/ark:/72163/1/0801/HSmk8KWbQjy6YCqnBrwYgA0.20250506T124135628531015Z.

19
https://ark.dasch.swiss/ark:/72163/1/0801/N1XIvGvYSBO1wODFfl0QjQL.20250506T135709210439294Z.

20 In the graphs given in the figures of this paper, instead of IRIs and actual local names of resources and predicates, reader-friendly identifiers are used.

21 See https://ark.dasch.swiss/ark:/72163/1/0801/GcXDoaZgRu6E2scC77h6gwz.

22
https://ark.dasch.swiss/ark:/72163/1/0801/t4g6hkPDRiSgUS1GijWHaAZ.20191030T15142755Z.

23
https://ark.dasch.swiss/ark:/72163/1/0801/7wCofGOOSzKc3HEo9bDz2wn.20191030T151036182Z.

24
https://github.com/dasch-swiss/sipi.

25
https://iiif.io/.

26 See http://www.dasch.swiss/dsp-app.

27 https://docs.dasch.swiss/2023.09.01/DSP-API/02-dsp-ontologies/knora-base/.

28 See https://ark.dasch.swiss/ark:/72163/1/0801/ucu2GlKXRSOUeDDzU5SmqgE.20160720T154544604Z.

29 See https://ark.dasch.swiss/ark:/72163/1/0801/7wCofGOOSzKc3HEo9bDz2wn.

30 See https://docs.dasch.swiss/latest/DSP-API/02-dsp-ontologies/knora-base/#text-with-standoff-markup.

31
https://www.mathjax.org/.

32 See https://ark.dasch.swiss/ark:/72163/1/0801/zcLJ81hdQFaH8Kq3kmcqCQ5.20240122T154712663976466Z.

33 See https://ark.dasch.swiss/ark:/72163/1/0801/zcLJ81hdQFaH8Kq3kmcqCQ5.

34
https://www.transkribus.org/.

35 See Alassi (2023) and https://sepidehalassi.github.io/textToRDFGraph/.

36 See https://ark.dasch.swiss/ark:/72163/1/0801/ja6xxc4WSrGQ_t9RgPTa_we.20240130T162825292560675Z.

37 See https://ark.dasch.swiss/ark:/72163/1/0801/ja6xxc4WSrGQ_t9RgPTa_we.

38 See https://ark.dasch.swiss/ark:/72163/1/0801/_KaGmqZLTyW9U9__vCUzLAc.20240130T185020456625Z.

39 See https://ark.dasch.swiss/ark:/72163/1/0801/ja6xxc4WSrGQ_t9RgPTa_we.20240130T162825292560675Z.

40 See footnote 19.

41
https://www.dasch.swiss/.

42
https://docs.dasch.swiss/latest/DSP-API/.

43
https://docs.dasch.swiss/latest/DSP-APP/.

44 See the representation of a letter Johann Bernoulli sent to Leibniz on Briefportal Leibniz here: https://leibniz-briefportal.adw-goe.de/letter/l35202.

45 See the representation of a letter Newton wrote to Henry Oldenburg on The Newton Project platform here: https://www.newtonproject.ox.ac.uk/view/texts/diplomatic/NATP00321.

46 See the representation of a letter from Haller Correspondence on the *HallerNet* platform here: Gerlach Adolph von Münchhausen an Albrecht von Haller, 5. Dezember 1751, in: Digitale Edition der Korrespondenz Albrecht von Hallers, *HallerNet* 2018–2023,https://hallernet.org/edition/letter/05889.

47 Accessible through https://bernoulli-euler.dhlab.unibas.ch/ and https://beol.dasch.swiss/.

48 A detailed description of the RDF-based versioning system is beyond the scope of this article and will be discussed in a future one. The documentation of the API’s versioning support is available here https://docs.dasch.swiss/latest/DSP-API/05-internals/design/principles/triplestore-updates/?h=version#versioning.
